# Journey to Perinatal Mental Health Services in Northern Ireland

**DOI:** 10.1192/bjo.2022.285

**Published:** 2022-06-20

**Authors:** Helen Connolly, Julie Anderson

**Affiliations:** 1 Western Health & Social Care Trust, Derry, United Kingdom; 2 Northern Health and Social Care Trust, Antrim, United Kingdom

## Abstract

**Aims:**

The case for perinatal psychiatry as a subspecialty is strong. In the context of perinatal mental illness consideration has to be given to; differences in presentation, the need to account for mother and baby and the risks associated with inadequate treatment. Specialist services improve outcomes, reduce risks and save money. Despite the government's agenda of preventative healthcare, service provision has been inequitable across the UK. Here we detail the journey towards the development of new Community Perinatal Mental Health Services in Northern Ireland (NI).

**Methods:**

In NI the first embers of a perinatal service were ignited by Dr Janine Lynch approximately 15 years ago when she established a small community perinatal team in Belfast Health and Social Care Trust (BHSCT). Her commitment and foresight regarding training inspired others, resulting in high levels of interest among trainees. From this grew a dedicated group of consultants committed to supporting service development across NI.

A multidisciplinary regional perinatal mental health forum was formed leading the development of a Northern Ireland Care Pathway in 2012. In partnership with women with lived experience, this forum led the bid for perinatal service development across the province.

**Results:**

Following years of campaigning the need for services was recognised in both the Bamford Review (2012) and RQIA Perinatal Review (2017). A commitment for funding for specialist teams, across all five health and social care trusts, was outlined in the Mental Health Action Plan in May 2020. Funding was finally approved in January 2021.

Significant work has gone into training to ensure there is a workforce ready to deliver services with focus on upskilling all professionals who deliver care to mums during the perinatal period. A competency framework has been developed to compliment this.

It is important to recognise the support and commitment of many members of the college Perinatal Faculty throughout this journey.

**Conclusion:**

Community perinatal mental health services are at an exciting juncture in NI. Each of the trusts have made a commitment to the development of services under the co-ordination of the Public Health Agency. Several have progressed to recruitment of key staff with the aspiration for services to go live before the end of the year. There will be an overarching, integrated approach, co-ordinated by the new Regional Perinatal Network.

As newly recruited consultants we look forward to working in partnership to address this long-standing health inequality and improve the outcome for women and their babies in NI.

